# Prevalence of Subjective Olfactory Dysfunction and Its Risk Factors: Korean National Health and Nutrition Examination Survey

**DOI:** 10.1371/journal.pone.0062725

**Published:** 2013-05-09

**Authors:** Woo Hyun Lee, Jee Hye Wee, Dong-Kyu Kim, Chae-Seo Rhee, Chul Hee Lee, Soyeon Ahn, Ju Hyun Lee, Yang-Sun Cho, Kun Hee Lee, Kyung Soo Kim, Si Whan Kim, Ari Lee, Jeong-Whun Kim

**Affiliations:** 1 Department of Otorhinolaryngology, Seoul National University Bundang Hospital, Seongnam; Seoul National University College of Medicine, Seoul, South Korea; 2 Medical Research Collaborating Center, Seoul National University Bundang Hospital, Seongnam, South Korea; 3 Department of Otorhinolaryngology-Head and Neck Surgery, Sungkyunkwan University School of Medicine, Seoul, South Korea; 4 Department of Otorhinolaryngology-Head and Neck Surgery, Kyung Hee University School of Medicine, Seoul, South Korea; 5 Department of Otorhinolaryngology-Head and Neck Surgery, Yonsei University College of Medicine, Seoul, South Korea; 6 Department of Otorhinolaryngology-Head and Neck Surgery, Hallym University College of Medicine, Anyang, South Korea; 7 Division of Chronic Disease Surveillance, Korea Centers for Disease Control & Prevention, Seoul, South Korea; Brigham & Women's Hospital, and Harvard Medical School, United States of America

## Abstract

**Background:**

Population-based studies for olfactory dysfunction are lacking. The aim of this study is to evaluate the prevalence of subjective olfactory dysfunction and its risk factors in the Korean general population.

**Methods:**

The data were obtained from the 2009 Korea National Health and Nutrition Examination Survey (KNHANES), which was a cross-sectional survey of non-institutionalized population all around the country (n = 10,533). All interviewees underwent medical interviews, physical examinations, endoscopic examination and blood/urine tests. Whether sense of smell has been normal or abnormal during the last 3 months was asked. Complete olfaction data were obtained from 7,306 participants and the participants were divided into normosmic and hyposmic group. Multivariate logistic regression analyses were performed to identify its risk factors.

**Results:**

The weighted prevalence of subjective olfactory dysfunction was 4.5%. Its increased prevalence was significantly associated with the increasing age for both men and women. In the multivariate analyses, low income (adjusted odds ratio [OR] = 1.43, 95% Confidence Interval [CI] = 1.01–2.03), habitual exposure to air pollutants (adjusted OR = 2.18, CI = 1.33–3.55), a history of hepatitis B (adjusted OR = 3.10, CI = 1.25–7.68), rhinitis (adjusted OR = 1.78, CI = 1.26–2.51) and chronic sinusitis (adjusted OR = 14.55, CI = 10.06–21.05) were risk factors of olfactory dysfunction.

**Conclusion:**

Our population-based study showed that olfactory dysfunction was quite prevalent and several risk factors were associated with impaired sense of smell. Given its prevalence, further researches for its prevention and management are required.

## Introduction

The loss of sense of smell decreases quality of life and may contribute to the failure in recognizing hazardous substances or unpleasant odors. [1] This problem can develop changes in appetite and this may make a nutritional problem especially in the elderly. Persons who have olfactory dysfunction are at an increased risk of danger from leaking gas, smoke, food spoilage, and pollution. [2] Although it is usually not a life-threatening or highly morbid condition, [3] it is important to recognize undiagnosed olfactory dysfunction and its etiology in order to prevent these possible complications. So far, population-based studies for olfactory dysfunction are lacking. The National Health Interview Survey (NHIS) of the United States including about 42,000 households showed that the prevalence of self-reported olfactory problems was 1.4%. [1] There were another two population-based studies in the Western countries, which were based on psychophysical tests. In the Epidemiology of Hearing Loss Study (EHLS) of individuals aged 53 to 97 years in Beaver Dam, 24.5% were found to have impaired sense of smell, [4] and the Skovde population-based study of the general Swedish adult population aged ≥20 years, 19.1% were found to have olfactory dysfunction. [5] However, there has been no large group study for olfactory impairment in the Asian population.

Although olfactory dysfunction needs to be objectively diagnosed and managed by clinicians, it is also important to know the prevalence of self-reported olfactory dysfunction which may determine whether they are likely to seek medical attention. [6] The current investigation was conducted to determine the prevalence of self-reported olfactory dysfunction in the large Korean population who were randomly selected. This study is also aimed to identify the risk factors of olfactory dysfunction through analyses of systematic medical questionnaires, endoscopic examinations and blood/urine tests.

## Materials and Methods

### Population

The Korea Centers for Disease Control and Prevention and the Korean Society of Otorhinolaryngology-Head and Neck Surgery have collaboratively collected medical history and clinical measurement data from a representative sample of the Korean population in the Korean National Health and Nutrition Examination Survey (KNHANES). The KNHANES 2009 data set was used, which included information on the presence of subjected olfactory dysfunction and endoscopic findings of the ear, nose and throat (ENT). A total of 12,722 individuals of 4,000 households were invited to participate in the KNHANES 2009. These participants were randomly selected from a panel to represent the population of South Korea. The panel was extracted by using a multistage clustered and stratified random sampling method that was based on the National Census Data. Among 12,722 selected individuals, 10,533 (82.8%) agreed to participate in the clinical examination. Analyses were conducted for the data from 7,306 participants who answered to the questionnaire for the presence of olfactory dysfunction. However, the other general characteristics of the subjects included were comparable with those of the subjects excluded. The average age of the 7,306 participants was 49.3±16.5 years (range, 20–95 years) and the ratio of male to female was 1∶1.32. Written informed consents were obtained from all the participants prior to the survey and approval for this research was obtained from the Institutional Review Board of the Seoul National University Bundang Hospital.

### ENT Evaluation, Medical History and Clinical Examination

The endoscopic ENT examinations and interview for otolaryngologic medical history were performed by trained ENT residents according to the standardized protocols. A total of 108 surveys were conducted by four survey teams during a time span of 27 weeks all over the country. Each survey team consisted of one ENT resident, three nurses, two interviewers, and one coordinator. Each survey was performed in a mobile examination unit vehicle at the pre-assigned places. A single visit was required for each participant to the examination unit vehicle. All the questionnaires, ENT examinations and blood/urine samplings were performed during the single visit. A total of 87 ENT residents from 45 teaching hospitals were recruited for performing ENT examinations. The Epidemiology Committee of the Korean Society of Otorhinolaryngology-Head and Neck Surgery verified the quality control of the survey.

The olfactory questionnaire was asked about whether the participants have had problems with the sense of smell during the past three months. The participants with a positive response were considered hyposmic and those with a negative response normosmic. Chronic rhinosinusitis was ascertained when nasal polyps were observed or more than one of the symptoms such as anterior/posterior nasal drip, nasal obstruction, facial pain/pressure, and olfactory dysfunction were present for more than 3 months (anterior/posterior nasal drip or nasal obstruction should be included as a presenting symptom). Otitis media was defined when there was a perforation in the tympanic membrane, cholesteatoma including a retraction pocket, or inflammation in the tympanic membrane with effusion. The diagnosis of rhinitis was made when they have had a history of rhinorrhea, sneezing, itching, and nasal obstruction for the last one year without cold symptoms. The deviation of the nasal septum was defined when an asymmetric displacement to one or both sides of the nasal cavities was observed after vasoconstriction of the nasal cavities.

Other medical histories were obtained by trained interviewers of the Korea Center of Disease Control and Prevention. A set of structured questionnaires were asked. The socioeconomic status (income, education, residency and occupation), smoking, drinking, a history of habitual exposure to air pollutants or chemical substances were asked and the participants were categorized. The study population was split into 6 subgroups by age. Body mass index was categorized into normal (<25 Kg/m^2^) and obese (≥25 Kg/m^2^). Residency was categorized into urban and rural areas according to the official address of the subjects. Home income was calculated by equivalized gross household income per month in each year and grouped into 4 quartiles. In the analysis, the subjects were divided into low (1^st^ and 2^nd^ quartiles) and high (3^rd^ and 4^th^ quartiles) groups. Subjects who graduated from middle or elementary school were considered to have a low level of education, and those from high school or college were considered to have a high level of education. Occupation was categorized by following the Korean Standard Classification of Occupations. The occupational categories were as follows: white collar (included managers, professionals, clerks, service/sales workers, unemployed, retired, students, and housewives) and blue collar (agriculture, forestry, fishery workers, craft and related trade workers, plant and machine operators and assemblers, and simple labor). Smoking status was classified into two categories: non-smoker (a person who has smoked below 5 pack smoking) and smoker (a person who has smoked more than 5 pack smoking). Alcohol consumption was assessed by asking the participants about their average frequency of alcoholic beverage consumption during the month prior to the interview.

Blood and urine samples were also tested. All the collected blood and urine samples were transported to a single designated laboratory (Neodin Medical Institute, Seoul, Korea) and analyzed according to their qualified standard protocols and equipments.

### Statistical Analyses

The data were analyzed with SPSS (version 18.0, SPSS Inc, Chicago, IL, USA), which is a software package that incorporates sample weights and adjusts analysis for the complex sample design of the survey. We used the KNHANES sampling weight variables, along with masked variance primary sampling unit and stratum variables. This adjustment allowed the estimation of the entire non-institutionalized Korean population from the sample. Survey sample weights were used in all the analyses. The chi-square test was used to compare the characteristics between normosmic and hyposmic participants. The independent *t*-test was used to analyze blood and urine test results. After performing univariate analyses of variables, the multiple logistic regression analysis was performed including variables with values of p<0.20 in the univariate analyses to calculate adjusted odds ratios (OR) and their 95% confidence interval (CI). After conversion of continuous variables to dichotomous variables (normal *vs.* abnormal) according to their normal laboratory range, blood and urine test results were also included in the multivariate logistic model. A p-value <0.05 was considered significant. Missing data were considered to be missing completely at random.

## Results

### Prevalence of Olfactory Dysfunction

**Figure 1:**
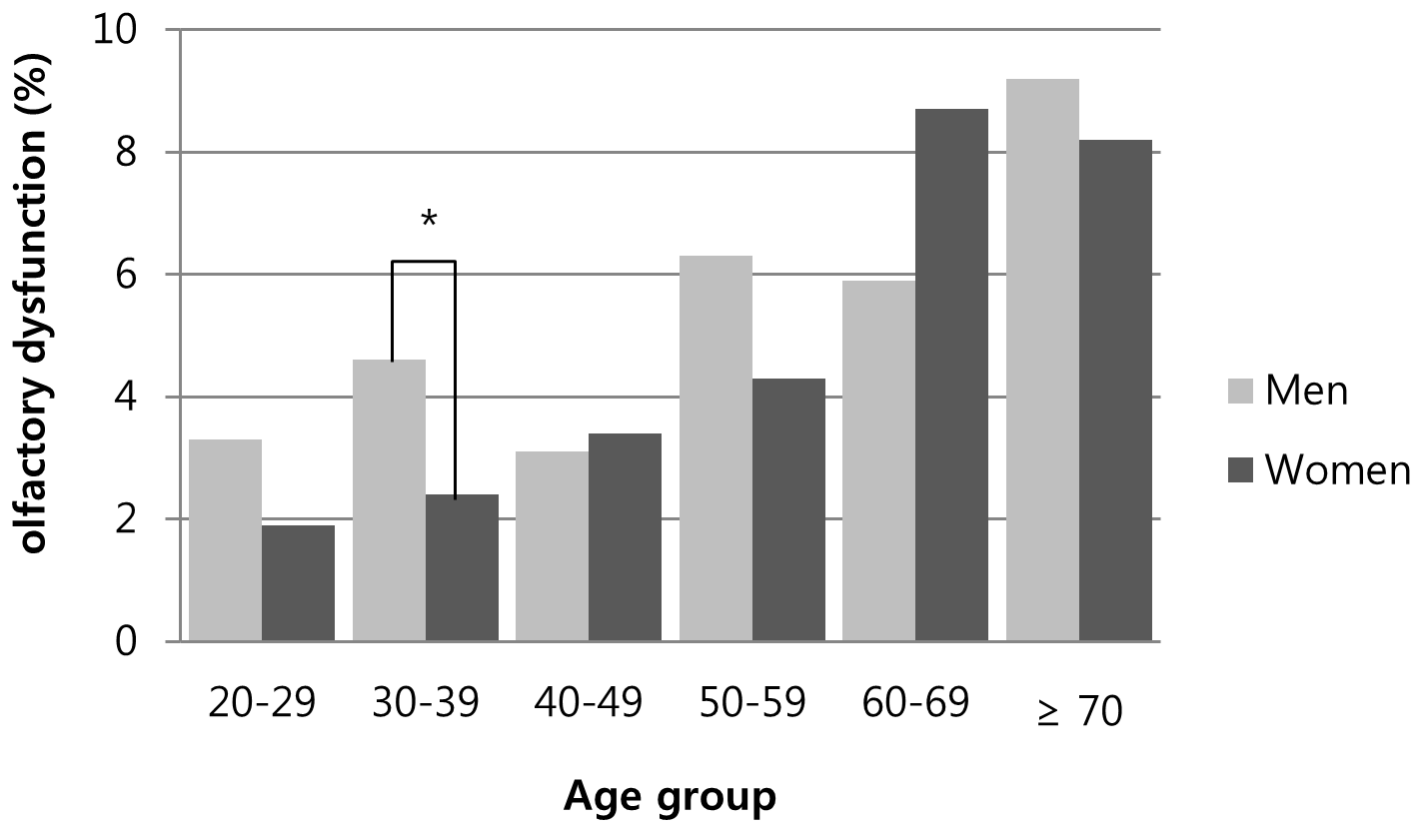
Prevalence of subjective olfactory dysfunction by age groups and genders. There were increasing tendency of olfactory dysfunction prevalence with increasing age in both genders, asterisk; p<0.05.

Of 7,306 participants, 360 (4.9%) had olfactory dysfunction and its weighted prevalence was 4.5%. The prevalence of olfactory dysfunction according to the general characteristics of the participants is shown in **Table 1**. The chi-square analyses showed that sex, occupation, residence, body mass index, habitual exposure to chemical substances, smoking and drinking did not affect the prevalence of olfactory dysfunction. A logistic regression analysis using olfactory dysfunction as the outcome variable and their age as a continuous variable after adjustment for sex showed a significant increasing trend of the prevalence with age (adjusted OR, 1.026; CI, 1.019–1.033; P value, <0.001). There was no significant difference was found between two genders in each age subgroup, except 30–39 years when the participants were categorized by a decade of age (**Figure 1**). The increased prevalence was significantly associated with lower education level (p<0.001), lower income (p<0.001) and habitual exposure to air pollutants (p = 0.001).

**Table 1:** Prevalence of olfactory dysfunction according to the general characteristics of KNHNES participants according to the presence of olfactory dysfunction (weighted for the multistage sampling design of KNHANES 2009).

		Olfactory dysfunction Weighted, % (SE)		
Characteristics	Unweighted total number	No	Yes	P value
**Overall**	7306	95.5 (0.4)	4.5 (0.4)	
**Age**				<0.001
20–29	936	97.4 (0.5)	2.6 (0.5)	
30–39	1407	96.5 (0.6)	3.5 (0.6)	
40–49	1473	96.7 (0.6)	3.3 (0.6)	
50–59	1233	94.7 (0.8)	5.3 (0.8)	
60–69	1203	92.6 (1.0)	7.4 (1.0)	
≥70	1054	91.4 (1.0)	8.6 (1.0)	
**Sex**				0.302
Male	3149	95.3 (0.5)	4.7 (0.5)	
Female	4157	95.8 (0.4)	4.2 (0.4)	
**Education**				<0.001
≤Middle school	2867	93.7 (0.7)	6.3 (0.7)	
≥High school	4380	96.3 (0.4)	3.7 (0.4)	
Missing	59			
**Income**				<0.001
≤50 percent	3240	94.2 (0.6)	5.8 (0.6)	
>50 percent	3983	96.4 (0.4)	3.6 (0.4)	
Missing	83			
**Occupation**				0.022
White collar	5287	95.9 (0.4)	4.1 (0.4)	
Blue collar	1937	94.5 (0.6)	5.5 (0.6)	
Missing	82			
**Residence**				0.469
Rural	5408	95.4 (0.5)	4.6 (0.5)	
Urban	1898	96.0 (0.6)	4.0 (0.6)	
**Body mass index**				0.601
<25 kg/m^2^	4926	95.4 (0.5)	4.6 (0.5)	
≥25 kg/m^2^	2331	95.7 (0.5)	4.3(0.5)	
**Air Pollutants**				0.001
Exposure	399	92.0 (1.6)	8.0 (1.6)	
No exposure	6884	95.7 (0.4)	4.3 (0.4)	
Missing	23			
**Chemical substance**				0.399
Exposure	230	94.2 (1.9)	5.8 (1.9)	
No exposure	7053	95.6 (0.4)	4.4 (0.4)	
Missing	23			
**Smoking**				0.709
<5 packs	4382	95.6 (0.4)	4.4 (0.4)	
≥5packs	2864	95.4 (0.5)	4.6 (0.5)	
Missing	60			
**Alcohol drinking**				0.519
≤4times/month	5746	95.6 (0.4)	4.4 (0.4)	
≥2times/week	1573	95.2 (0.7)	4.8 (0.7)	
Missing	23			

SE, standard error; Air pollutants, habitual exposure to air pollutants in workplace; Chemical substance, habitual exposure to chemical substances in workplace.

The chi-square analyses from the medical history data set demonstrated that several medical conditions were associated with olfactory dysfunction (**Table 2**). Among ENT diseases, chronic sinusitis, rhinitis and chronic otitis media were associated with olfactory dysfunction. In the univariate analyses, hypertension, thyroid diesease, low HDL (high density lipoprotein) cholesterolemia, asthma, hepatitis B, arthritis, osteoporosis, gastric cancer and cataract were also associated with olfactory dysfunction. In the independent t-test analyses of blood and urine test results, olfactory dysfunction was associated with decreased levels of blood HDL cholesterol and urine creatinine and increased levels of blood GOT (glutamic oxaloacetic transaminase), BUN (blood urea nitrogen) and lead (**Table 3**).

**Table 2:** Prevalence of olfactory dysfunction according to the medical conditions of KNHANES participants.

		Olfactory dysfunction Weighted, % (SE)		
Characteristic	Unweighted total number	No	Yes	P value
**ENT diseases** †				
Chronic rhinosinusitis	402	68.8 (3.3)	31.2 (3.3)	<0.001
Rhinitis	1696	91.6 (0.9)	8.4 (0.9)	<0.001
Chronic otitis media	261	90.5 (2.4)	9.5 (2.4)	0.001
Septal deviation	3090	95.2 (0.5)	4.8 (0.5)	0.405
**Cardiovascular disease**				
Hypertension	1513	93.8 (0.7)	6.2 (0.7)	0.001
Stroke	143	93.7 (1.8)	6.3 (1.8)	0.236
Angina	137	94.1 (2.0)	5.9 (2.0)	0.398
Arrhythmia	292	95.5 (1.1)	4.5 (1.1)	0.963
**Metabolic disease**				
Diabetes mellitus	573	95.7 (0.8)	4.3 (0.8)	0.850
Thyroid disease	254	89.9 (2.7)	10.1 (2.7)	0.001
Hyperlipidemia	574	94.4 (1.1)	5.6 (1.1)	0.205
Low-HDL cholesterolemia	1802	94.0 (0.9)	6.0 (0.9)	0.014
**Pulmonary disease**				
Tuberculosis	400	95.5 (0.4)	4.5 (0.4)	0.833
Asthma	224	87.9 (3.7)	12.1 (3.7)	0.001
COPD	43	94.7 (2.7)	5.3 (2.7)	0.749
Bronchiectasis	25	90.0 (7.5)	10.0 (7.5)	0.296
**Gastro-intestinal disease**				
Gastric ulcer	452	95.3 (1.1)	4.7 (1.1)	0.800
Hepatitis B	86	86.4 (4.6)	13.6 (4.6)	0.001
**Neuromuscular disease**				
Rheumatoid arthritis	148	93.6 (2.3)	6.4 (2.3)	0.336
Arthritis	967	92.4 (1.1)	7.6 (1.1)	<0.001
Osteoporosis	539	90.6 (1.5)	9.4 (1.5)	<0.001
**Neoplastic disease**				
Gastric cancer	49	86.3 (6.2)	13.7 (6.2)	0.016
Hepatic cancer	11	94.7 (10)	5.3 (5.3)	0.870
Colon cancer	17	89.4 (8.3)	10.6 (8.3)	0.260
**Others**				
Depression	277	93.8 (1.6)	6.2 (1.6)	0.195
Anemia	473	95.2 (1.0)	4.8 (1.0)	0.751
Atopic dermatitis	161	93.0 (2.1)	7.0 (2.1)	0.121
Cataract	660	92.5 (1.1)	7.5 (1.1)	<0.001

HDL, high density lipoprotein; COPD, chronic obstructive pulmonary disease;

† All ENT diseases were diagnosed by ENT residents.

**Table 3:** Comparison of blood/urine tests between normosmic and hyposmic participants.

	Olfactory dysfunction weighted (SE)		
Variable	No	Yes	P value
**Blood test**			
Fasting glucose (mg/dL)	96.90 (1.30)	97.39 (1.24)	0.705
HbA1_C_ (%)	7.30 (0.23)	7.37 (0.21)	0.761
Insulin (µIU/mL)	9.89 (0.32)	9.56 (0.31)	0.300
Total cholesterol (mg/dL)	186.14 (2.20)	186.60 (2.17)	0.835
HDL cholesterol (mg/dL)	52.28 (0.88)	50.03 (0.88)	0.010
Triglyceride (mg/dL)	135.70 (7.80)	143.98 (7.76)	0.289
LDL cholesterol (mg/dL)	110.96 (4.79)	106.35 (4.71)	0.337
GOT (IU/L)	22.41 (0.88)	24.35 (0.85)	0.037
GPT (IU/L)	22.49 (1.73)	23.80 (1.71)	0.451
BUN (mg/dL)	14.17 (0.25)	14.81 (0.25)	0.013
Vitamin D (ng/mL)	17.74 (0.40)	17.76 (0.43)	0.953
Alkaline phosphatase (IU/L)	221.99 (3.43)	222.19 (3.32)	0.954
Lead (µg/dL)	2.49 (0.14)	2.89 (0.14)	0.006
Mercury (µg/L)	5.10 (0.41)	5.80 (0.41)	0.094
Cadmium (µg/L)	1.08 (0.07)	1.09 (0.07)	0.936
**Urine test**			
Creatinine (mg/L)	158.54 (6.03)	143.99 (5.86)	0.017
Sodium (g/day)	131.69 (4.00)	134.51 (3.94)	0.482
Cotinine (ng/mL)	445.86 (64.07)	393.55 (63.01)	0.415

HbA1c, hemoglobin A1c; HDL, high density lipoprotein; LDL, low density lipoprotein; GOT, glutamic oxaloacetic transaminase; GPT, glutamic pyruvic transaminase; BUN, blood urea nitrogen.

### Multivariate Analyses of Risk Factors

In multivariate logistic regression analyses, the effect of each variable was adjusted for all the other variables (**Table 4**). In reference to the youngest subgroup aged 20–29 years, the odds of having olfactory dysfunction were significantly higher in the older subgroups aged 50–59 years (adjusted OR, 2.09; 95% CI, 1.16–3.77), 60–69 years (adjusted OR, 2.58; 95% CI, 1.31–5.08) and 70 years or older (adjusted OR, 3.17; 95% CI, 1.93–7.14). Lower income (adjusted OR, 1.43; 95% CI, 1.01–2.03), habitual exposure to air pollutants (adjusted OR, 2.18; 95% CI, 1.33–3.55), a history of hepatitis B (adjusted OR, 3.10; 95% CI, 1.25–7.68), rhinitis (adjusted OR, 1.78; 95% CI, 1.26–2.51) and chronic sinusitis (adjusted OR, 14.5; 95% CI, 10.06–21.05) were significantly associated with self-reported olfactory dysfunction. However, all of the blood and urine test results were not associated with the olfactory dysfunction.

**Table 4:** Adjusted odds ratio for the association between olfactory dysfunction and risk factors.

Exposure	Adjusted OR	CI	P value
Age group			
20–29	1.00		
30–39	1.08	0.60–1.95	0.792
40–49	1.31	0.73–2.37	0.360
50–59	2.09	1.16–3.77	0.014
60–69	2.58	1.31–5.08	0.006
≥70	3.71	1.93–7.14	<0.001
Income (≤50 percent)	1.43	1.01–2.03	0.042
Education (≤Middle school)	0.67	0.43–1.04	0.075
Occupation (Blue collar)	1.21	0.86–1.69	0.256
Air pollutants	2.18	1.33–3.55	0.002
Hypertension	0.94	0.63–1.41	0.796
Arthritis	0.99	0.62–1.58	0.968
Osteoporosis	1.32	0.81–2.13	0.259
Asthma	1.84	0.81–4.20	0.143
Depression	0.85	0.38–1.87	0.688
Atopic dermatitis	1.16	0.50–2.69	0.713
Thyroid disease	1.71	0.83–3.50	0.142
Cataract	1.06	0.69–1.63	0.784
Gastric cancer	1.44	0.34–6.08	0.612
Hepatitis B	3.10	1.25–7.68	0.014
Low HDL cholesterolemia	1.41	0.97–2.05	0.067
Rhinitis	1.78	1.26–2.51	0.001
Chronic otitis media	1.34	0.74–2.43	0.328
Chronic sinusitis	14.55	10.06–21.05	<0.001
HDL (<40 mg/dL)	0.82	0.62–1.10	0.192
GOT (>40 IU/L)	1.82	0.86–3.81	0.113
BUN (>24 mg/dL)	1.90	0.92–3.93	0.080
Lead (>10 µg/dL)	1.07	0.38–3.04	0.888
Mercury (>15 µg/dL)	1.20	0.42–3.34	0.730

OR, odds ratio; CI, confidence interval; HDL, high density lipoprotein; GOT, glutamic oxaloacetic transaminase; BUN, blood urea nitrogen; each exposure variable is adjusted for all other variables in the table; After conversion of continuous variables to dichotomous variables (normal *vs.* abnormal) according to their normal laboratory range, blood and urine test results were included in the multivariate logistic model.

## Discussion

This is the first large population-based study reporting the prevalence of subjective olfactory dysfunction in Asia. The weighted prevalence of subjective olfactory dysfunction in the Korean population was 4.5%. In other words, it is equivalent approximately to 1.7 million (according to the current census population of South Korea) Korean adults. The prevalence was obtained from a national survey, KNHANES 2009, which randomly recruited participants.

Prevalence studies based on psychophysical or semi-objective tests can provide more objective information. The prevalence evaluated by psychophysical tests in the Epidemiologic Hearing Loss Study was much higher than that obtained by questionnaires. The gap between the prevalence of self-reported olfactory dysfunction and that of semi-objective olfactory dysfunction increased with age. [4] This suggests that elderly persons would be likely to be unaware of their impaired sense of smell. The sensitivity of subjective olfactory dysfunction was low (20%) but the specificity was very high (94%). Furthermore, the sensitivity of subjective olfactory dysfunction decreased with age. [4] Another study also showed that self-reported olfactory dysfunction had low sensitivity (43.9%) and high specificity (85.4%). [5] These findings suggest that many people with an impaired sense of smell may be unaware of their olfactory problem. Given the low sensitivity of self-reported questionnaire, the present study shows that olfactory dysfunction is not a negligible disease because the self-reported prevalence was as high as 4.5% in our study. Therefore, it is important to identify the prevalence of self-reported olfactory dysfunction to make the patients seek medical attention, initiate medical care and prevent possible complications.

The present study also identified several risk factors associated with olfactory dysfunction. Age, socioeconomic status, environment and some medical conditions were likely to affect the impaired sense of smell. The odds of having olfactory dysfunction increased with age in both men and women in the present study. A previous large population study in the U.S. [1] showed similar results in that the prevalence of self-reported olfactory dysfunction was 1.99%, 2.56%, and 4.6% for subgroups aged 55–64 years, 65–74 years, and 75 years or older, respectively. Several mechanisms may be involved in age-related olfactory dysfunction. Long-lasting injuries to the olfactory mucosa such as various co-morbidities or exposure to environmental olfactory toxicants have been speculated to lead to the replacement of olfactory mucosa with respiratory epithelium. [7] Atrophy of the olfactory bulb and tract occurs in elderly people as the number of glomeuli and mitral cells decreases with age.[8]–[10] Structural magnetic resonance imaging showed an age-associated volume loss in the mesial temporal lobe which is important for olfactory processing. [11].

With regard to gender, some studies performed in the Western countries showed that olfactory dysfunction was more prevalent in men than in women. [12], [13] However, our nationwide study demonstrated that its prevalence was not statistically different between men and women. The different result may be caused by differences in the study design and population. Given that our study samples were selected in a random fashion to represent the whole national population and asked by ENT residents who have been trained for olfactory disorders, there is unlikely to be sexual differences in olfactory dysfunction in Koreans.

As previously reported, [4] lower income was also found to be associated with olfactory dysfunction in our study. People with higher income may have better access to health care or have healthier lifestyles than people with lower incomes. [14] Environmental factors such as an exposure to chemicals or air pollution can also be factors associated with olfactory dysfunction. KNHANES showed that the habitual exposure to air pollutants in their workplaces was significantly associated with the higher prevalence of olfactory dysfunction. This finding suggests that we should also involve olfactory toxicity when the effects of air pollution in the workplaces on our health and quality of life are evaluated. On the other hand, a questionnaire about the habitual exposure to chemicals in their workplaces could not show its detrimental effects on olfaction in our study even if there were some reports for olfactory impairment caused by an occupational exposure to airborne industrial chemicals.[15]–[18] Tobacco smoking might also be a factor affecting the sense of smell. However, its harmful effects on olfaction are under controversy [5] and we could not observe its significant effects. Some studies have reported adverse effects of smoking on olfactory function, [19], [20] whereas other studies reported no effect on olfaction. [21], [22] Even if our cross-sectional study failed in identifying the olfactory toxicity of chemicals and smoking, further studies are required after several related factors such as exposure amount and duration are more strictly controlled.

Airway diseases can also affect the sense of smell. Although upper airway diseases such as chronic sinusitis and rhinitis were significantly associated with olfactory dysfunction, lower airway diseases such as bronchial asthma and chronic pulmonary airway disease showed no association. It is well known that hyposmia, dysosmia and dysgeusia are common symptoms in hepatitis. [23], [24] Improvement in olfactory acuity was inversely related to the plasma-bilirubin level and directly related to the plasma-retinol-binding-protein level in acute hepatitis. [25] To our knowledge, this is the first large population-based study showing that a history of hepatitis B is associated with olfactory dysfunction although its mechanism is unclear.

The present study also has some limitations. First, olfactory function was evaluated by questionnaires instead of psychophysical tests. However, as ENT questionnaires were asked by trained ENT residents, the decision of olfactory dysfunction may be clinically quite relevant. Secondly, as this is neither a longitudinal study nor a strictly controlled experimental study, the causal relationship of the risk factors with olfactory dysfunction may be inconclusive. Nevertheless, the results may be reliable because this is a nationwide population-based study all around the country. Thirdly, the control of the diseases which may relate to olfactory dysfunction may pose influences on results. However, whether those diseases have been well controlled or not could not be evaluated in this study.

In conclusion, our population-based study showed that 4.5% of Korean population suffered from olfactory dysfunction. In addition to ENT diseases and medical conditions, old age, low income and habitual exposure to air pollutants were associated with an increased risk of impaired sense of smell. Given the quite high prevalence of olfactory dysfunction in Korea, further researches for its prevention and management are required.
